# Paper-based chemometer device for the estimation of α-amylase—a biomarker for pancreatitis†

**DOI:** 10.1039/d4ra03804e

**Published:** 2024-08-05

**Authors:** Bethuel Daurai, Manashjit Gogoi

**Affiliations:** a Department of Biomedical Engineering, North-Eastern Hill University Shillong Meghalaya India bethueldaurai@gmail.com manash.aec@gmail.com

## Abstract

Pancreatitis is a life-threatening inflammatory disease of the pancreas. In 2019, 34.8 out of 100 000 people suffered from acute pancreatitis globally. In humans, the level of α-amylase increases three times the normal value during pancreatitis. α-Amylase is an enzyme that hydrolyses α-1,4 glycosidic bonds of starch. In this study, we investigated a novel distance-based sensing method. We exploited the existing starch triiodide method, where the blue colour of starch-triiodide fades away and becomes colourless when α-amylase breaks the starch chain at the α-1-4 glycosidic bond. A hydrophilic channel was made on paper using a simple laser printer to create hydrophobic barriers. This channel was impregnated with starch triiodide, where α-amylase can turn it colourless. This distance covered by the change in colour is directly proportional to the concentration of α-amylase in a sample. Simulated samples with different concentrations of porcine α-amylase and pancreatin were used for testing using the developed paper-based chemometer device. The paper-based chemometer device was also tested with artificial blood serum with different concentrations of α-amylase. The *R*^2^ of this device was found to be 0.9905, and the accuracy of the device when compared with a 2-chloro-4-nitrophenyl-α-d-maltotrioside method was found to be 95.54% with a sensitivity of 0.131 U L^−1^ mm^−1^. Correlation test also showed that the paper-based chemometer device for α-amylase can be used as a testing device for artificial blood serum. This is a preliminary investigation that shows promising results. The chemometer devices stored in air-tight packets at 4–8 °C in a refrigerator did not lose the colour intensity until day 90 and retained an accuracy of 94.5%. However, the device needs to be evaluated in clinical settings prior to using it for measuring α-amylase in patients.

## Abstract 2

We show the working of a paper-based chemometer device (PCD). Starch triiodide coated on the channel discolours upon the addition of α-amylase. The discoloured distance is directly proportional to the concentration of α-amylase.

## Graphical abstract

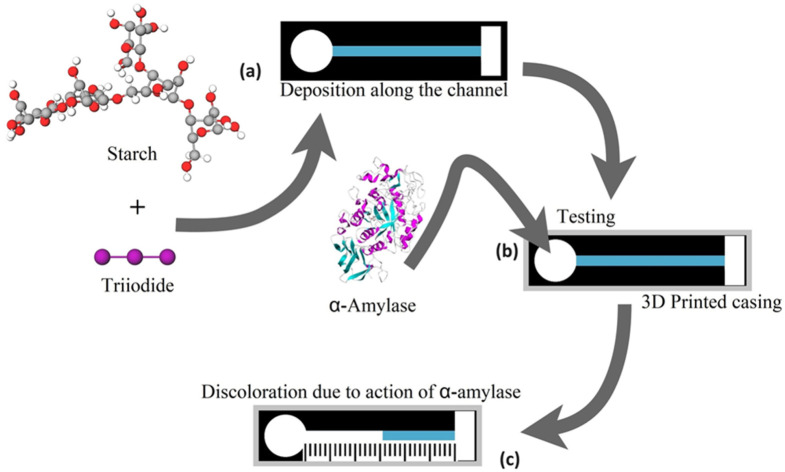

## 1. Introduction

Paper-based devices have become a successful platform for the estimation of analytes, with potential applications in industries such as healthcare, environmental monitoring, food safety, and drug development.^1,2^ Dipstick-based devices are widely used owing to their advantages, such as low cost, portability, and ease of use, in resource-limited settings.^3,4^ The principle behind these devices is based on the capillary action of fluids,^5^ where fluids move in the paper channel due to the attraction between the fluid and paper fibres.^6–8^ The distance travelled by the fluid is proportional to the analyte concentration in the sample, allowing for quantitative analysis.^9,10^ The simplicity and accessibility of these devices make them ideal for use in resource-limited settings where traditional laboratory equipment and methods may not be feasible.^11^ There are various inexpensive methods for the development of channels on paper. Some of them include wax printing, screen printing, photolithography, laser cutting, three-dimensional printing, *etc.*^12–14^ These fabrication techniques make the paper an alternative for making cheaper lab-on-chip devices for analysis purposes in the food and beverage industries and diagnostic purposes in the medical sector.^15,16^ The use of paper-based materials also makes these devices environmentally friendly and easily disposable.

Paper-based devices can be easily transported and stored, making them ideal for field testing in remote and rural areas where laboratory facilities are hardly available.^17,18^ The devices do not require any specialised training or equipment, and the results can be easily interpreted without the need for complex data analysis.^19^ They have also demonstrated excellent performance in various chemical analyses, including the detection of glucose, cholesterol, and protein biomarkers in biological fluids.^20–22^ These devices have also been used for environmental monitoring, such as the detection of heavy metal ions in water samples, and food safety testing, such as the detection of pathogens in food products.^23–25^ There is ongoing research focused on improving the sensitivity and selectivity of distance-based paper-based microfluidic devices using new materials and technologies, such as nanoparticles and biosensors, to enhance the detection capabilities of these devices.^26–29^

α-Amylase measurement contributes to the timely identification of pancreatitis. Pancreatitis is an inflammation of the glandular organ pancreas situated posterior to the stomach.^30^ The global burden of acute pancreatitis depends on the prevalence of the disease. In 2019, 34.8 persons out of 100 000 suffered from acute pancreatitis globally.^31^ In North America, 52 persons suffered from pancreatitis out of 100 000, whereas the highest incidence was recorded in Eastern Europe, which stood at 79.6 in 100 000 people and was also prevalent in low-income regions, such as South-East Asian countries as well as South and Central American nations. As per the revised Atlanta classification, α-amylase serves as the principal biomarker for pancreatitis detection.^32^

In this study, a paper-based chemometer device (PCD) developed using a laser printer is investigated for measuring α-amylase, a biomarker for pancreatitis. Hydrophilic channel saturated with starch triiodide exhibits a profound blue colour due to the triiodide forming bonds with the alpha-1,4 linkages of large polysaccharides.^33,34^ The channel undergoes a colour change from blue to colourless upon the introduction of α-amylase from one end.^35^ The measurement of the distance at which the colour transitions from deep blue to colourless across the channel is utilised to determine the concentration of α-amylase. The developed PCD was used to determine α-amylase for both simulated samples and artificial blood serum. All the results in this study are shown for the artificial blood serum.

Existing studies suggest the use of the same principle of starch triiodide and other techniques for the detection of α-amylase in various biological fluids.^36–40^ Some devices use colourimetric, fluorometric, liquid crystal, surface plasmon resonance, mechanic, and electrochemical probes.^37,41–43^ The main distinction lies in the fact that these earlier studies required a supporting electronic device, while the PCD we developed is standalone, and α-amylase estimation can be performed with naked-eye visualisation. This PCD device will not replace the existing methods of detection but will supplement them by aiding in ruling out pancreatitis in rural areas with community healthcare centres and can also aid in ruling out clinics and emergency medicine.

## 2. Experimental

### 2.1 Reagents and materials

Potato starch pure, pancreatin 4NF extrapure and α-amylase ex. porcine pancreas were procured from SRL (P) Ltd, Taloja, India. Iodine, A. R. and potassium iodide were procured from HIMEDIA, Mumbai, India. Grade 4 Whatman filter paper was obtained from GE Healthcare, Buckinghamshire, United Kingdom. Pancreatin 4NF extrapure has an α-amylase activity of 100 U mg^−1^ and can be used to hydrolyse starch when prepared in 0.2 M phosphate buffer at pH 6.8. 2-Chloro-4-nitrophenyl-α-d-maltotrioside (CNPG3) obtained from Euro Synergy Bio, Chennai, India, was used as the reference test to compare the data produced by the PCD. Artificial blood serum (ABS) was procured from Biochemazone, Alberta, Canada, for the validation of PCD. To make the casing for the PCD, poly lactic acid filament was purchased from eSun3D, Shenzhen, China.

### 2.2 Preparation of reagents

Lugol's solution was prepared using 1 g of potassium iodide (KI) and 0.5 g of iodine mixed in 10 mL of deionized water. Then, 50 μL of this prepared Lugol's solution was added to the 20 mL of 2% starch (400 mg in 20 mL) solution.

α-Amylase stock solutions were prepared from both porcine α-amylase and pancreatin by dissolving their lyophilised powders in a phosphate buffer solution of pH 6.8 and ABS. Stock solutions of both materials were prepared, with concentrations ranging from 70–420 U L^−1^. Amylase (pancreatin) was added to the ABS and PBS to prepare different solutions with amylase concentrations ranging from 70–420 U L^−1^. The viscosity of both PBS and ABS (containing α-amylase) solutions was measured using an Ostwald's viscometer to evaluate the impact of viscosity on the flow of the fluid in the PCD. The test was performed on the PCD channels by dropping 100 μL each of α-amylase solution.

### 2.3 Design and fabrication of microfluidic channels

The PCD channels were designed using a computer-aided design (CAD) software tool. Dimensions are optimised using the trial and error methods described in a previous study.^44^ The channels were developed by depositing hydrophobic toner ink on the desired part and letting it seep into the paper's pores, as described in previous studies.^12,45–47^ This was done using a laser printer (Canon MF3010) and toner ink without any modification to the printer. This method was adopted by Ghosh *et al.*, although the same procedure did not produce a flawless hydrophobic channel on our printer.^47^ In our study, we used a laser printer to print patterns on paper. The large-sized Whatman filter papers were cut down to ISO A4 size to be able to print with the paper. The printed pattern was then heated to 185 ± 5 °C in an oven for 6 hours so that the ink could seep into the pores of the paper, forming the barrier. This temperature and time were both optimised using the trial-and-error method. A case was designed using CAD software for encasing the paper channel and was 3D printed.

The stability of a PCD depends on uniformity in the pore sizes and quality of the paper used. Uniformity in pore sizes ensures the fluid retention capacity of the device and the flow rate of the fluid.^48,49^ Whatman filter papers were used to ensure the quality of paper, fluid retention capacity and constant flow rate of fluid. Both Grade 1 (pore size approximately 11 μm) and Grade 4 (pore size approximately 20–25 μm) Whatman filter papers were investigated for the development of the chemometer. However, Grade 4 Whatman filter paper was found to be suitable for our device due to the higher retention capacity of reagents, higher flow rate of fluid and hence less reaction time. Moreover, printing hydrophobic material on Grade 4 filter paper is easier because of its larger pore size.

### 2.4 Deposition of reagents on the channel and characterisation

**Fig. 1:**
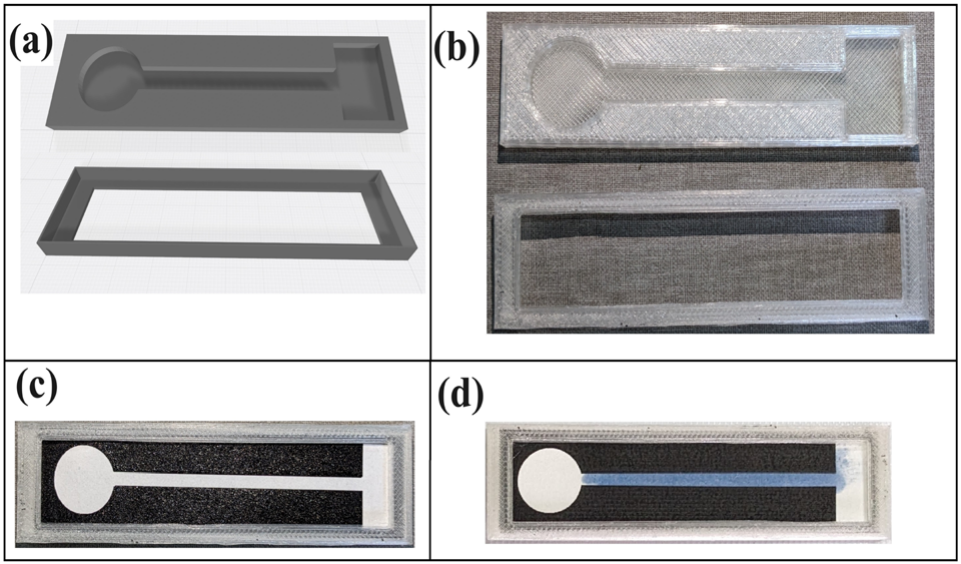
(a) CAD design of the casing containing the cavities. (b) 3D printed structure of the design with polylactic acid as the material. (c) Fitting of the paper-based channel in the casing. (d) Intense blue starch triiodide coated on the channel.

The starch triiodide solution was deposited on the channel, as shown in Fig. 1(d). The strips of PCD were drop-coated with an equal amount of starch triiodide until both sides of the surface turned deep blue. The channels were dried at room temperature (22–25 °C) for about 2–3 hours.

UV-visible spectroscopy (Inkarp Sican 2000) was used for the spectrum scan. The scan was done to determine the peak absorbance of starch triiodide and to observe if the peak reduced with the action of α-amylase. The estimation of α-amylase is well established using the starch triiodide method with the UV-visible spectroscopic method. Fourier transform infrared spectroscopy (FTIR) characterisation was done for starch triiodide, α-amylase, and the reaction. The scan was taken from the paper surface of the PCD channels. This was done to determine the presence of the functional group in the reaction. The concentration of α-amylase was taken at 420 U L^−1^ for the characterisation of both UV-visible spectroscopy and FTIR.

### 2.5 Statistical testing and validation using the standard method

The calibration curve was created with various concentrations of α-amylase ranging from 70 to 420 U L^−1^. The experiment was performed three times, and the mean values were used to prepare the calibration curve.

The CNPG3 method adopted by Euro Synergy Bio was used as the standard method for the validation and comparison of the PCD. The CNPG3 method is a good assessment for microanalysis system.^50^ This CNPG3 method works with UV-visible spectroscopy, which has linearity up to 2000 U L^−1^. The accuracy of the PCD was determined by estimating the concentrations, *i.e.*, 70, 140, 210, 280, 350, and 420 U L^−1^ of α-amylase in ABS. All the concentrations were tested with both PCD and CNPG3 for accuracy using the following equation. The accuracy was calculated by subtracting the percentage error from 100%.1
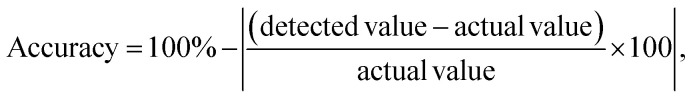
where the detected value is the value detected by the device and the actual value is the value detected by applying the CNPG3 method.

To determine the storage stability of the device, the fabricated PCDs were stored in the following conditions: (i) room temperature open; (ii) room temperature sealed in an airtight packet; (iii) 4–8 °C refrigerated open, and (iv) 4–8 °C refrigerated sealed airtight packet and used to determine α-amylase in simulated samples after 90 days.

To test the reproducibility of our device, the correlation of the 3 sets of tests shown in Fig. 5 was performed to determine the linear response of the three sets of tests. ANOVA single factor was also done on the 3 sets of PCD data. The null hypothesis is as follows: the 3 sets of data are similar or follow the same pattern. The alternate hypothesis is as follows: there is no similarity among the 3 sets of data.

A test of correlation and a *t*-test (two samples assuming equal variance) were performed between the PCD and the CNPG3 method to determine the linear response test and determine the usability of the PCD in place of the CNPG3 method. The data were normalised before running the test because both had the *X*-axis as an arbitrary value. In the paired *t*-test, the null hypothesis was that the PCD and CNPG3 methods were similar and could be interchanged in the determination of the two methods. The alternative hypothesis was that the two methods were completely different and could not be used interchangeably in the estimation of α-amylase.

## 3. Results and discussion

### 3.1 Design and fabrication of microfluidic channels

The filter paper was printed three times using toner ink to create a thick deposit of the substance. Special care should also be taken while making the second and third print layers because misalignment can distort the channel widths. It was heated for 6 hours. If the heating duration exceeds six hours, the hydrophobic material diffuses into the channel intended for hydrophilicity. Furthermore, if the duration is less than five hours, the seepage of the hydrophobic substance does not reach the reverse side of the printed paper. This allows for the escape of fluid through the border. Fig. 1 shows the design of the PCD and the printed PCD.

A case was developed to maintain the hydrophilic part of the PCD strips elevated. This case prevents unwanted fluid leakage from the PCD. Fig. 1(a) shows the 3D design and the printed case for the PCD. Creality Ender 3 S1 (3D Printer) was used to print the case, and the material used for printing was polylactic acid. The case is reusable and biodegradable if chosen for disposal.

**Fig. 2:**
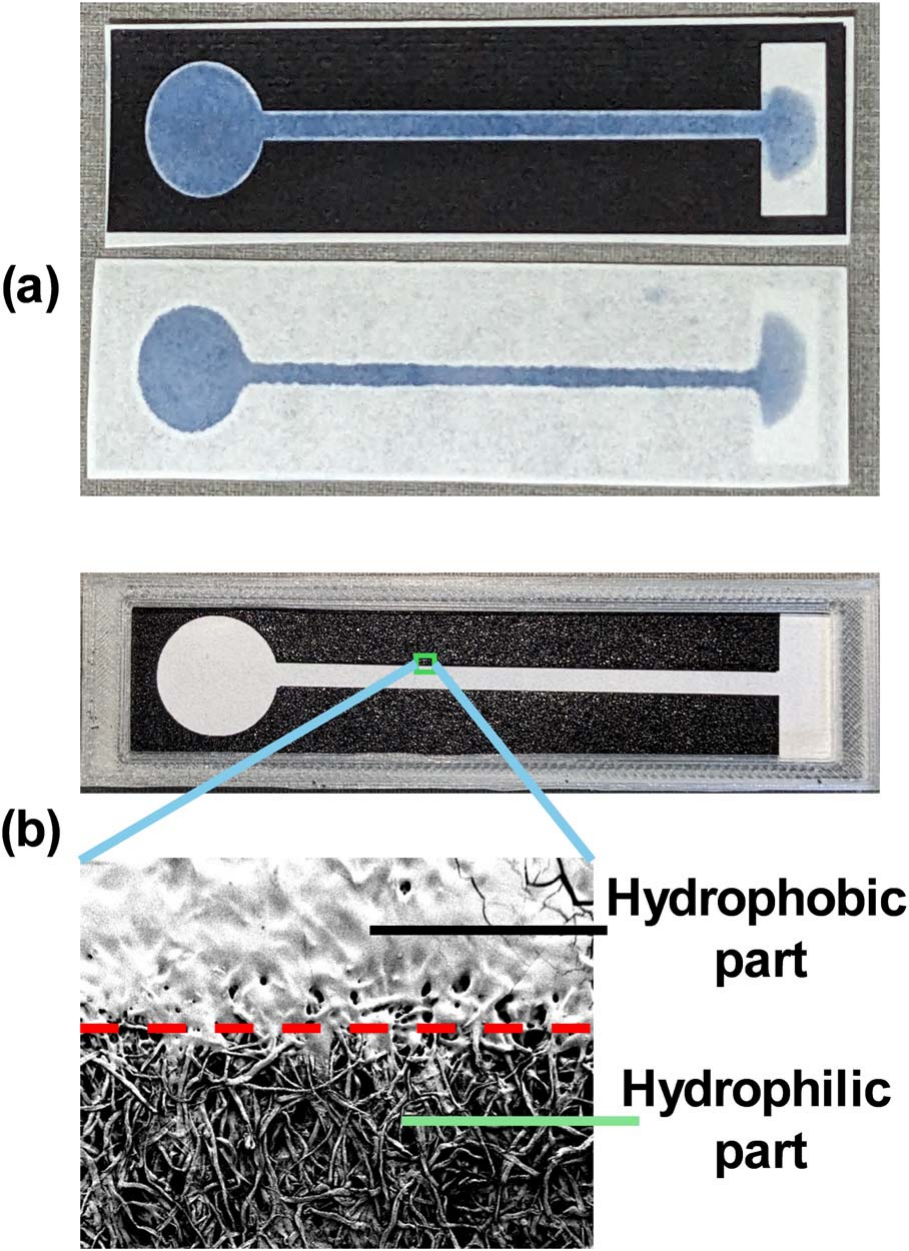
(a) Top view of the channel and bottom view of the channel showing that the channel was formed and that there was no leakage of any fluid. (b) Scanning electron microscopy image of the hydrophobic barrier showing the clogging of the pores in the Whatman filter paper.

In Fig. 2(a), photographs of the channels are shown from both the top and bottom of the channels to show that the channel was formed without leakage. The toner ink, although not visible on the bottom, seeps to the bottom, forming a hydrophobic barrier. Another optional layer could be printed on the opposite surface of the paper with a mirror image. This step is optional because the uniform printing may be compromised because of human error, which leads to the narrowing of the channels. Starch triiodide was used as a coloured solution to show that the channels were intact and to check for any leakage along the channel. A slight overspread of the hydrophobic material was observed along the border of the channel and the hydrophobic barrier. This can be avoided by reducing the heating time by about half an hour of the mentioned time. However, in this application, the heating time was not reduced.

In Fig. 2(b), a microscopic image was captured using a field emission scanning electron microscope (FE SEM Zeiss Sigma) to observe whether the pores of the channels had been clogged and had gained hydrophobicity with the toner ink. Similar results have also been observed in previous studies.^13,47^ This shows that the pores were all filled with the melted toner material. Magnification was taken 100 times with a 100 μm size of the view, and the size of the pores was approximately 23.5 μm when measured with Image J software (Grade 4 Whatman filter paper usually has a pore size of 20–25 μm). A slight overspread of hydrophobic material can also be observed at the edge of the barriers, as shown in the photograph in Fig. 2(a).

### 3.2 Deposition of reagents on the channel and characterisation

In the preparation of the sample, iodine (I2) and potassium iodide (KI) react to form potassium triiodide (KI3), as shown in eqn (2). This reaction results in the formation of triiodide ions (I_3_^−^), as shown in eqn (3).

2 I_2_ + KI → KI_3_

3 I_2_ + I^−^ → I_3_^−^

As long as iodine molecules and iodide ions are present, triiodide ions are continuously produced.^51^ The triiodide ions are responsible for the brown hue of the solution, which is caused by the complex triiodide ions. The triiodide ions react with starch to form starch triiodide, which is blue in colour.^52^ This reaction is represented in the following equation:

4 Starch + I^3−^ → starch triiodide.

This range was chosen to cover at least 3 times the normal range from the biological reference value. Now, this starch triiodide is coated to the channel, as shown in Fig. 1(d). The drying is performed at room temperature because, with heat, the blue colour dissipates into colourless by itself because of the breaking of the α-1,4 glycosidic bond. The final channel looked bright and light blue, which was easily distinguishable from the naked eye.

The volume of the starch triiodide was calculated based on the volume of fluid retained in the channel. Considering the channel as a cuboid structure, the amount of fluid retained is length × breadth × height. The thickness of the paper is 210 μm which is considered as the height. Therefore, the volume is 37.8 mm^3^, which is equivalent to 37.8 μL.

**Fig. 3:**
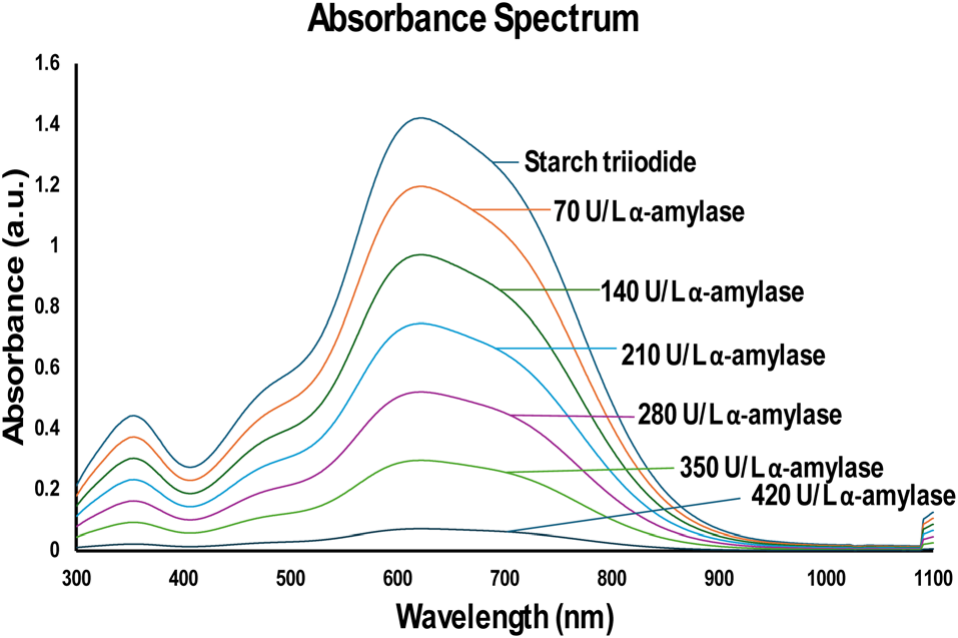
UV-vis spectrum analysis revealed that the prepared starch triiodide exhibited a peak absorbance at 600 nm, with a subsequent decrease in absorbance with an increase in the concentration of α-amylase from 70 to 420 U L^−1^.

A UV-vis spectrophotometric analysis of starch triiodide and the reaction of starch triiodide with α-amylase are shown in Fig. 3. Starch triiodide of the same concentration deposited in the PCD was used, and it showed peak absorbance at 600 nm. A concentration of 70–420 U L^−1^ of α-amylase was taken and mixed with starch triiodide at the ratio of 1 : 10, and the reading was taken, as shown in Fig. 3. The mixture was incubated at 37 °C for 15 minutes. This shows that a serial dissemination of the peak occurs. This shows that the activity of α-amylase hydrolyses the α-1,4 glycosidic bond, and the intense blue turns colourless.

**Fig. 4:**
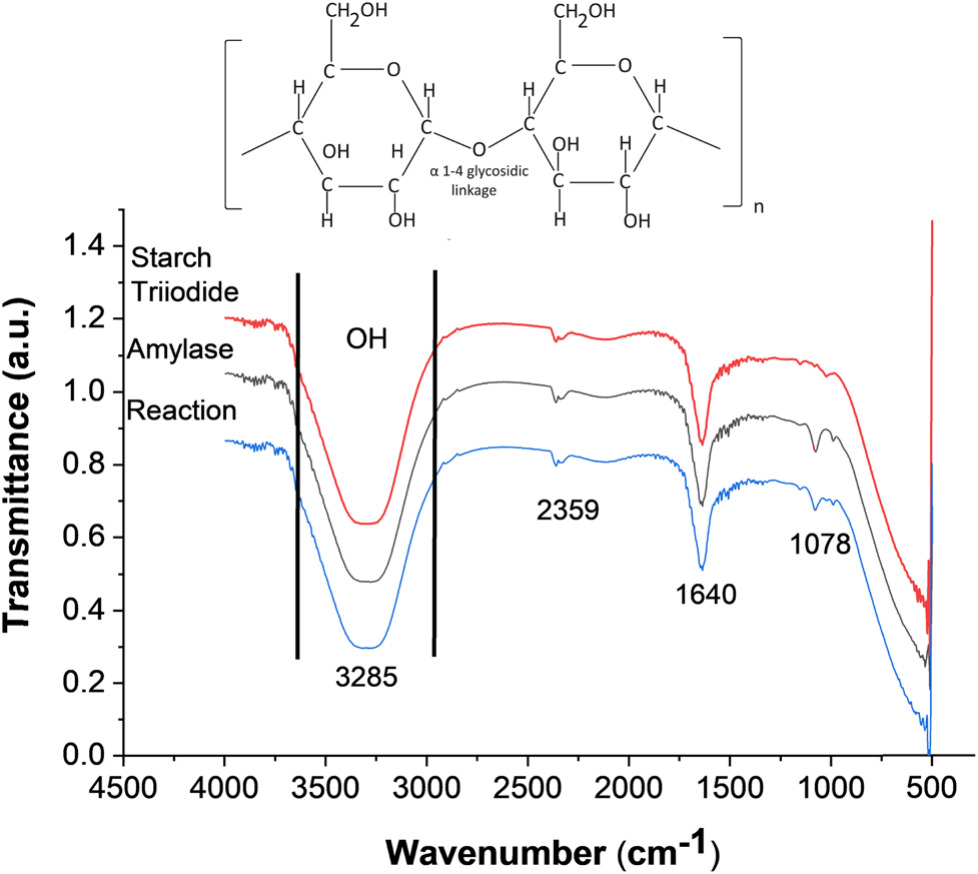
FTIR spectra of starch triiodide and amylase and their hydrolysis reactions.

Fig. 4(a) illustrates the FTIR spectra of α-amylase, starch triiodide, and the reaction, which were in the PCD channel. The FTIR reading was taken directly from the paper channel. A molecular structure of starch is also given to show various bonds and their presence in the FTIR spectra. The stretched peak pattern around 3000–3600 cm^−1^ in wave number shows the presence of an OH bond in all the three samples. This shows that the bond vibrates at that specific wavenumber, and all the three compounds had –OH (hydroxyl group) as their functional group. Starch usually contains an acetal group. The bending vibration at 1078 cm^−1^ is present in α-amylase, which is also observed in the reaction solution with starch triiodide. This bend signifies the presence of α-amylase in the reaction. The other smaller bending peaks are also visible at the 1640 cm^−1^ and 2359 cm^−1^ wavenumbers. Fig. 1 (ESI)† shows the FTIR of the serial dilution of α-amylase from 70–420 U L^−1^. The FTIR of these concentrations was taken from the solutions used in the absorbance spectrum and not directly in the PCD, as depicted in Fig. 4. As the amylase concentration decreases, there are observable shifts and changes in the intensity of the peaks, indicating alterations in the chemical environment and interactions between the molecules.

### 3.3 Statistical testing and validation using the standard method

After the formation of hydrophilic channels with hydrophobic barriers, the channels were used to estimate the concentration of α-amylase using the starch triiodide method. Viscosity was first tested for both the PBS- and ABS-based materials. In this method, the time taken by the fluid through a capillary is also measured. The viscosity is measured using eqn (5):^53^5
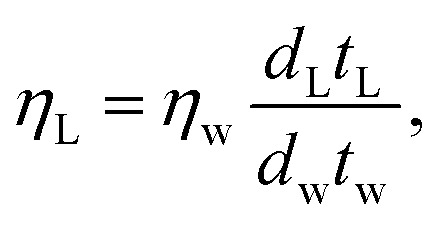
where *η*_L_ is the viscosity of the measured liquid, *η*_w_ is the viscosity of the reference liquid, *d*_L_ is the density of the given liquid, *d*_w_ is the density of the reference, *t*_L_ is the time taken by the test liquid to flow through the capillary and *t*_w_ is the time taken by the reference liquid to flow through the capillary. The viscosity obtained for the PBS base sample prepared in the laboratory was slightly more viscous than that of the ABS (PBS sample = 1.110925 centipoise, ABS = 1.007677 centipoise). Because the viscosity was only marginally different in the samples, there was no significant difference in the flow rate of the fluids.

**Fig. 5:**
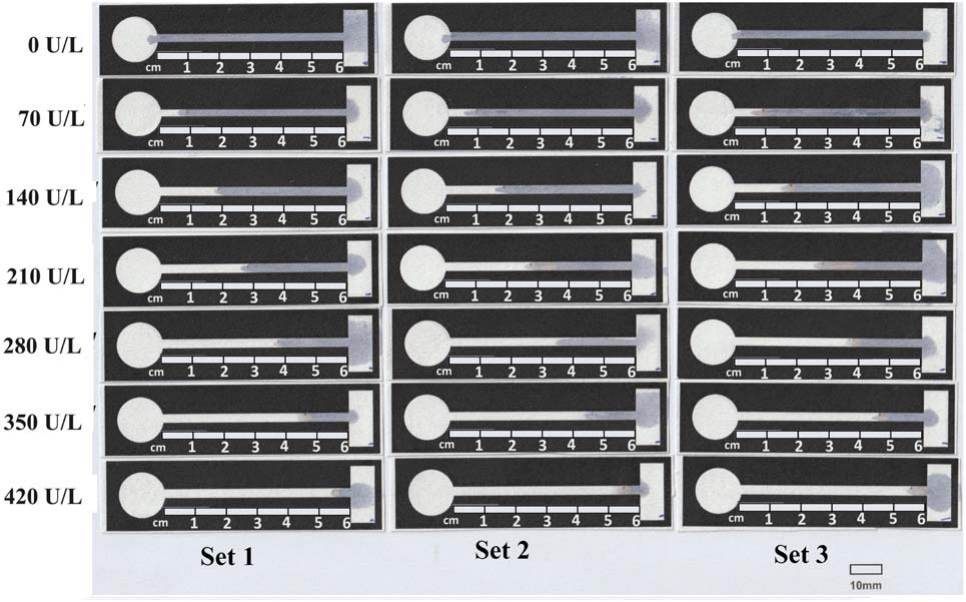
Test carried out for various concentrations of α-amylase from 70 to 420 U L^−1^.

Fig. 5 shows a scanned photograph of the tests performed. All the dried test strips were attached to a sheet of paper ISO A4. A scale of 10 mm was printed on a sheet of paper for reference. As enzyme α-amylase starts its action, the α-1,4 glycosidic bond in the starch starts breaking, losing its blue colour. This leads to the channels turning from blue to white. The discolouration because of the breaking of the α-1,4 glycosidic bond spreads according to the concentration of the enzyme. All the data derived from Fig. 5 are tabulated in Table 1.

**Table 1:** Data derived from Fig. 5, which is plotted in the calibration curve and the scattered plot

Conc. (U L^−1^)	D1 (mm)	D2 (mm)	D3 (mm)	Average (mm)	SD
0	0	0	0	0	0
70	5.882	7.061	10.084	7.68	2.17
140	17.984	17.482	18.151	17.87	0.35
210	26.219	33.284	36.302	31.94	5.18
280	36.472	34.034	37.311	35.94	1.70
350	46.386	43.868	45.883	45.38	1.33
420	54.79	54.453	55.127	54.79	0.337

However, ascorbic acid present in the blood serum is known to interfere with the starch triiodide complex. It readily oxidises the iodine in the starch triiodide complex. Dutta *et al.* described neutralizing ascorbic acid in the sample before it could be tested using the starch triiodide method.^36^ Potassium iodate was used to pretreat this blood serum to neutralise the effect of ascorbic acid on the starch triiodide complex.^54–56^ Potassium iodate was made from potassium iodide and iodine, and the pH was adjusted with sodium hydroxide. Potassium iodate is mixed with the blood before it can be introduced to the PCD.

**Fig. 6:**
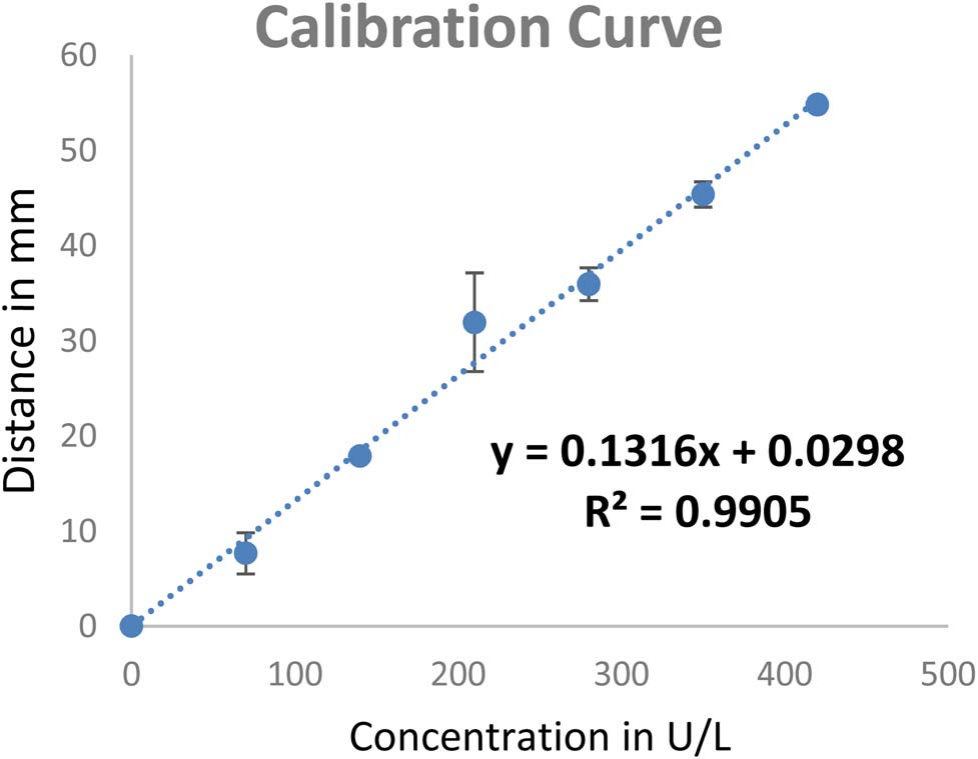
Calibration curve concentration against distance derived from Fig. 5.

The calibration curve obtained showed that the *R*^2^ value obtained was 0.9905 with a sensitivity of 0.131 U L^−1^ mm^−1^ (shown in Fig. 6). The determination of the accuracy of the PCD was compared with that of the standard CNPG3 method. The obtained accuracy was 95.54%. This shows that PCD as a POC device for the determination of α-amylase concentration is possible.

In the stability test, it was observed that the PCDs stored at room temperature without the seal lost their colour after 3 days, and the sealed packet PCDs stored at room temperature also lost their colours after 30 days. Both the PCDs stored at room temperature were not tested because they lost the intense blue colour, which shows that the α-1,4 bond has already been broken because of temperature fluctuations. The PCDs stored at 4–8 °C in refrigerated but not sealed packets also lost colour because of moisture. The PCDs stored at 4–8 °C refrigerated sealed airtight packets did not lose the colour intensity after 90 days. Once more, an accuracy test was conducted in comparison to the CNPG3 method, which produced a result of 94.5%.

**Fig. 7:**
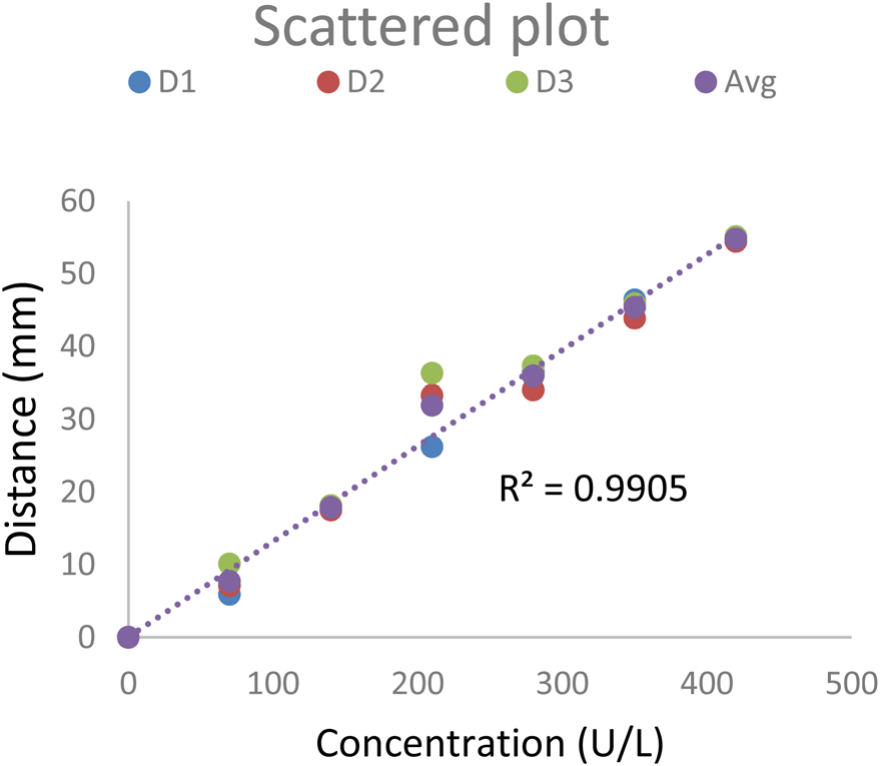
Scatter plot of the data. The trend line is taken for the average of the three sets of tests.

The Pearson's correlation value of 0.982 for the three repeated tests with the PCD suggests that they have a strong positive linear relationship. Table 1 (ESI)† shows the standard deviation (SD) with the precision level of the PCD at various data points. Fig. 7 shows the scattered plot of the three sets of data and the regression line of the average values corresponding to Fig. 6. This high correlation value also suggests the PCD's high reproducibility in quantifying the α-amylase in the ABS. An ANOVA single factor for the same three sets of data was performed with a significance level of 0.05. The *F* value was 0.0233, indicating no significant difference between the groups, and the *P* value was 0.976, indicating that there was no rejection of the null hypothesis (all three sets of data were similar).

A *t*-test (two samples assuming equal variance) was performed with *α* 0.05 significance value. The values were first normalised, and the *P* value found was 0.89, showing that there was no significant difference between PCD and the CNPG3 method and that there was no significant difference in the mean. Hence, we failed to reject the null hypothesis.

The Pearson correlation for the normalised data for both methods was 0.985, showing a very strong positive linear relation between the two methods. The *p*-value for the Pearson correlation between the normalised PCD data and the normalised standard data is approximately 5.11 × 10^−5^. The very small *p*-value indicates that the observed correlation is not due to random chance but because of the high accuracy of the PCD.

The 95% confidence interval for the Pearson correlation coefficient between the normalised PCD data and the normalised standard data is approximately [0.899, 0.998]. The interval further reinforces the strong positive correlation observed between the PCD's measurements and the standard, indicating that the developed PCD is likely to provide consistent and reliable results comparable to the standard.

## 4. Conclusion and future scope

The advantages of the developed prototype PCD include biodegradability and low cost because paper is used as the main component. The casing can be reused or disposed of without any harm to the environment because it is made up of polylactic acid. There are some challenges in the fabrication of hydrophobic channels. To obtain a thick deposition and even distribution of the hydrophobic pattern on the paper, the printer software can be tweaked, or multiple prints can be made on the same surface.

For the test performed with the ABS, the PCD determined the concentration of the enzyme α-amylase with an accuracy of 95.54%. The *R*^2^ value achieved was 0.9905. Colour change by dissipation of blue colour to colourless was observed in all the concentrations of the enzyme with different distances in mm. The paired *t*-test and Pearson correlation also suggest the usability of this PCD as a method to determine α-amylase. This device is applicable in testing applications as a POC device.

One of the future aspects of this device is to replace this starch triiodide with other reagents based on colourimetry. Some of the reagents that can be used are based on nanotechnologies, such as copper/gold nanocluster peroxidase and chitosan-tripolyphosphate core coated with a starch–iodine shell.^57,58^

Although the elevation of the enzyme α-amylase in serum during pancreatitis is of a shorter duration (three days), it can still be utilised to diagnose the condition at the onset of symptoms. The developed PCD device is working effectively in determining α-amylase in ABS. The PCD developed is a working prototype and will be useful for testing α-amylase in the low-income region of the world where the incidents of acute pancreatitis.^31^ This PCD can be used in rural areas as a first-line test for screening pancreatitis, which can even be helpful in emergency medicine (provided the community healthcare centre is equipped with refrigerators). However, the PCD was tested using only ABS. The results of this PCD need to be validated with real samples prior to its clinical application.

## Data availability

The datasets generated and/or analyzed during our study titled “Paper-based chemometer device for the estimation of α-amylase—a biomarker for pancreatitis” will be made available upon reasonable request. The datasets include all raw data, processed data, and ESI† related to the experiments conducted. Interested researchers can obtain the data by contacting the corresponding author. We are committed to ensuring transparency and reproducibility in our research and are happy to share our data to facilitate further scientific inquiry. Additionally, we have provided ESI† in a separate file.

## Conflicts of interest

There is no conflict of interest.

## Supplementary Material

RA-014-D4RA03804E-s001
