# Supplementary material for: Paper-based chemometer device for the estimation of α-amylase—a biomarker for pancreatitis
Source: RSC Adv. 2024 Aug 5;14(34):24365–72. doi: 10.1039/d4ra03804e (PMC11298970; doi:10.1039/d4ra03804e)
Supplement: RA-014-D4RA03804E-s001 [file RA-014-D4RA03804E-s001.pdf]

Supplementary Information (SI) for RSC Advances.
This journal is © The Royal Society of Chemistry 2024

# Paper-based Chemometer Device for the estimation of α-amylase, a biomarker for Pancreatitis

Bethuel Daurai[a], Manashjit Gogoi *[b]

a. Department of Biomedical Engineering, North-Eastern Hill University, Shillong, Meghalaya, India. bethueldaurai@gmail.com
b. Department of Biomedical Engineering, North-Eastern Hill University, Shillong, Meghalaya, India. manash.aec@gmail.com

This supplementary information contains data is obtained from Figure 5 in Table 1. The distance covered by the colour change of each chemometer device is entered in the table. This data is then used to make the calibration curve and the scattered plot in the main manuscript. It also contains the serial dilution Fourier Transform Infrared Spectroscopy (FTIR) of the various concentration of amylase in starch triiodide solution. Figure 1 (supplementary file) shows FTIR of the serial dilution of α-amylase from 70-420 U/L. The FTIR of these concentrations were taken from solutions used in the absorbance spectrum and not directly in the chemometer device and done in Figure 4 (of the manuscript). As amylase concentration decreases, there are observable shifts and changes in the intensity of the peaks, indicating alterations in the chemical environment and interactions between the molecules.

Table 1: Data derived from the Figure 5 in the manuscript which is plotted in the calibration curve and the scattered plot

| **Conc (U/L)** | **D1 (mm)** | **D2 (mm)** | **D3 (mm)** | **Average (mm)** | **SD** |
|---|---|---|---|---|---|
| **0** | 0 | 0 | 0 | 0 | 0 |
| **70** | 5.882 | 7.061 | 10.084 | 7.68 | 2.17 |
| **140** | 17.984 | 17.482 | 18.151 | 17.87 | 0.35 |

| | | | | | |
|---|---|---|---|---|---|
| **210** | 26.219 | 33.284 | 36.302 | 31.94 | 5.18 |
| **280** | 36.472 | 34.034 | 37.311 | 35.94 | 1.70 |
| **350** | 46.386 | 43.868 | 45.883 | 45.38 | 1.33 |
| **420** | 54.79 | 54.453 | 55.127 | 54.79 | 0.337 |

Table 1 data is obtained from Figure 5. The distance covered by the colour change of each chemometer device is entered in the table. This data is then used to make the calibration curve and the scattered plot in the main manuscript.

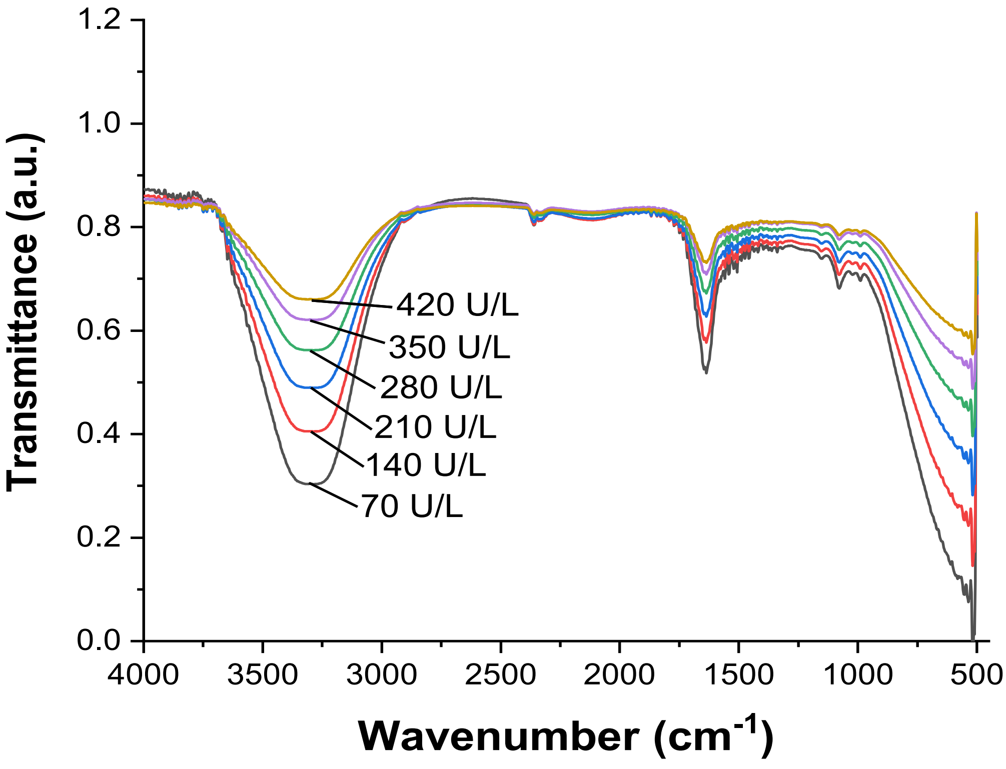

Figure 1: FTIR of the serial dilution of the amylase concentration in the reaction with starch triiodide.
